# Best Practices to Design, Plan, and Execute Large-Scale Federated Analyses—Key Learnings and Suggestions from a Study Comprising 52 Databases

**DOI:** 10.1055/a-2710-4226

**Published:** 2025-10-30

**Authors:** Theresa Burkard, Montse Camprubi, Daniel Prieto-Alhambra, Peter Rijnbeek, Marta Pineda-Moncusi

**Affiliations:** 1 Nuffield Department of Orthopaedics, Rheumatology and Musculoskeletal Sciences (NDORMS), Centre for Statistics in Medicine, University of Oxford, Oxford, United Kingdom; 2 Synapse, Carrer de la Diputació, Barcelona, Cataluña, Spain; 3 Department of Medical Informatics, Erasmus University Medical Centre, Rotterdam, The Netherlands

**Keywords:** OMOP, federated analyses, best practice, OHDSI, EHDEN

## Abstract

**Background:**

Federated network studies allow data to remain locally while the research is conducted through the sharing of analytical code and aggregated results across different health care settings and countries. A large number of databases have been mapped to the Observational Medical Outcomes Partnership (OMOP) Common Data Model (CDM), boosting the use of analytical pipelines for standardized observational research within this open science framework. Transparency, reproducibility, and robustness of results have positioned federated analyses using the OMOP CDM within the European Health Data and Evidence Network (EHDEN) as an essential tool for generating large-scale evidence.

**Objectives:**

We conducted large-scale federated analyses involving 52 databases from 19 countries using the OMOP CDM. In this *State-of-the-Art/Best Practice* article, we aimed to share key lessons and strategies for conducting such complex, large multidatabase analyses.

**Results:**

Meticulous planning, establishing a strong community of collaborators, efficient communication channels, standardized analytics, and strategic division of responsibilities are essential. We highlight the benefits of network engagement, cross-fertilization of ideas, and shared learning. Further key elements contributing to the study's success included an inclusive, incremental implementation of the analytical code, timely engagement of data partners, and community webinars to discuss and interpret study findings.

**Conclusion:**

We received predominantly positive feedback from data custodians about their participation, and included input for further improvements for future large-scale federated network studies from this shared learning experience.

## Background and Significance

Federated analytics describes a framework that facilitates the collaboration of multiple organizations and individuals while complying with diverse data protection and sharing legislation. In such a framework, the analyses are run by the local organization. Therefore, the data stay within their local environment, and only analytical code and results (i.e., anonymous aggregated data) are shared between collaborators. When combined with the use of a Common Data Model (CDM; i.e., transforming the data into a standard structure), the same analytical code can be executed across the network. 1 2

A major player within the federated analyses space is the Observational Health Data Sciences and Informatics (OHDSI) community, which uses the Observational Medical Outcomes Partnership (OMOP) CDM. The OHDSI 2024 annual report states that around 550 databases worldwide have been mapped to OMOP. 3 Previous efforts in the OHDSI community conducted network analysis with up to 26 databases. 4 In Europe, more than 200 databases have mapped their data to OMOP between 2019 and 2024 as part of the European Health Data and Evidence Network (EHDEN). 5 Many of these databases have organized themselves into national nodes, where different OMOP databases (i.e., various research institutions) within a country join forces. To date, 14 countries have established a national node in Europe. 6 Furthermore, standardized analytic packages allow fast evidence generation across different health care settings and countries with transparent, reproducible, and robust analyses. 7 8 Regulators have also become aware of the potential of federated analytics in health care data using OMOP, with a notable example being the European Medicines Agency's (EMA) DARWIN EU (Data Analysis and Real World Interrogation Network) initiative. 9

By having assessed a multinational issue (i.e., drug shortages), we provided an example of the potential of federated analytics and the use of OMOP, as well as training to individuals and institutions who were newly introduced into this framework. Our large-scale federated network study across the EHDEN network and consortium partners included 52 databases across 19 countries. 10 In this *State-of-the-Art/Best Practice* article, we aimed to describe how a large-scale study was conducted from planning to execution, offering a practical approach based on our recent experience. By sharing key learnings and actionable recommendations, we seek to support other teams interested in conducting federated studies.

## Nomenclature

Federated analysis, and especially the OHDSI community, has adopted a specific terminology. To provide context for the following sections, we define the key terms as follows:

- Database: A combination of multiple information from different data sources stored and linked datasets, structured in a specific format.
- Data partner: A data partner (or data custodian) has an OMOP-mapped database.
- OMOP CDM standardization: Structural harmonization and mapping of local terminologies used to describe clinical conditions, medications, procedures, and other health care concepts to the standardized vocabularies adopted by the OMOP CDM. This semantic standardization is essential to ensure that the same analytical code can be executed consistently and comparably across all data sources. 2 11
- Standardized analytics: Tools and R packages designed to be executed with databases previously mapped to OMOP, which contains standard analytic code and produces standard analytic output. 7 8

## The Study

In 2023, we launched a call across the EHDEN network to participate in our study ( Table 1 ), where we shared our study question: The annual incidence and prevalence of medicines with suggested shortages according to the EMA's drug shortages catalogue 12 between 2010 and 2023 ( *n* = 18 plus 39 key alternatives). 10 As a secondary objective, we aimed to describe annual trends among the users of these medicines, including their demographics (age and sex), and the medicine's indication of use, duration, and dosage (to assess trends in use prior, during, or after shortages). Our study contained 52 databases from 19 countries and >617 million people.

**Table 1:** Timeline sketch of our real-world evidence study containing 52 databases in 19 countries

Month	−2	−1	0	1	2	3	4	5	6	7	8	9	10	11	12	13	14	15	16	17	18
Protocol drafting																					
Call for participation																	FD				
Call for protocol feedback						D															
Protocol registration a																					
Database specifications b																					
Ethical approval hand-in														D	D		D	FD			
Feasibility code									D						D		D	FD			
Analyses 1 code												D			D		D	FD			
Analyses 2 code														D	D		D	FD			
Webinar preparation																					
First webinar																					
Preprint preparation																					
Preprint publication																					
Webinar preparation																					
Second webinar																					
Preprint update																					
Manuscript submission																					

Abbreviations: D, deadline; FD, final deadline.

a Protocol registration with the “HMA-EMA Catalogues of real-world data sources and studies.”

b Database-specific information required from participating data partners included short database descriptions, database acronyms (approved display names for publications), and full names of the database.

**Fig. 1:**
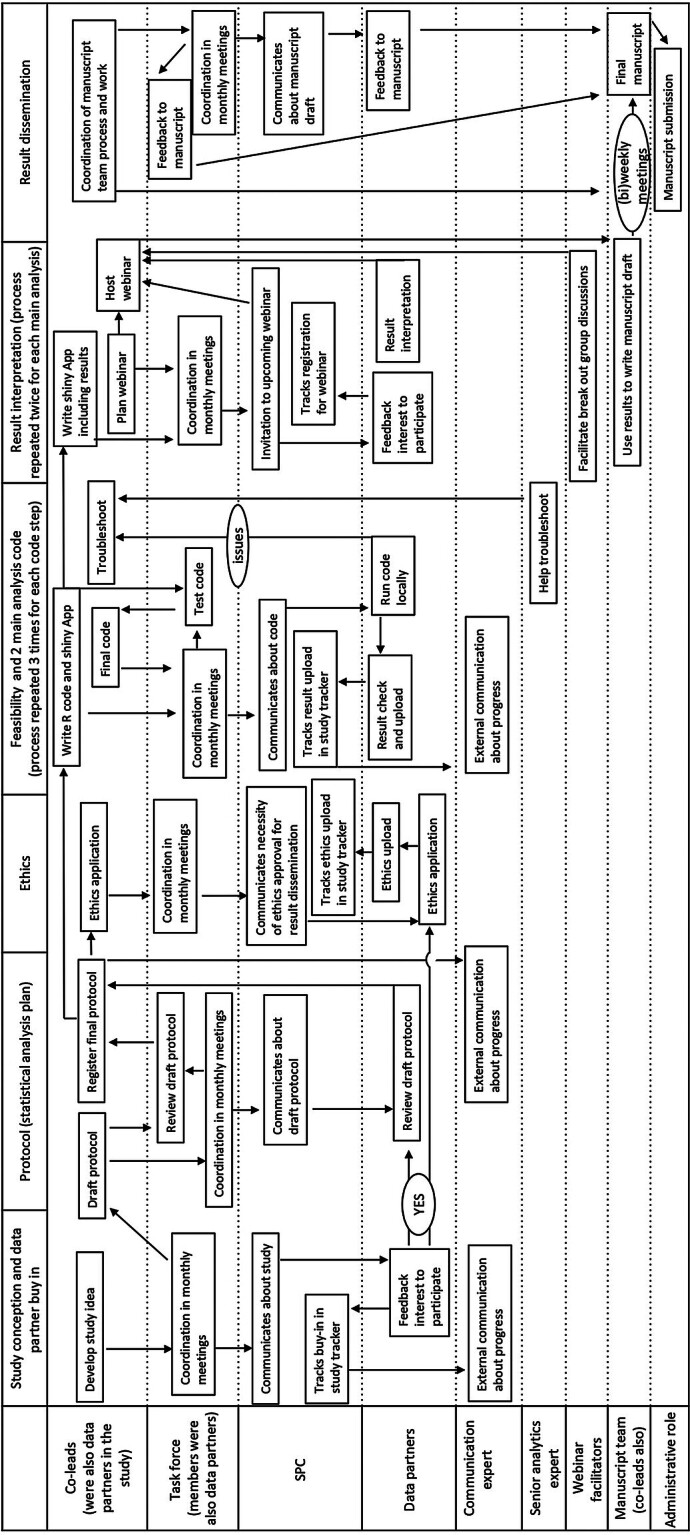
Process flow diagram of how we designed, planned, and executed our large-scale federated analytics network study across domains and involved players. The analytical code included feasibility, incidence-prevalence, and characterization of incidence-prevalence drug users; for the latter two, we held a webinar to discuss results. The manuscript team was created after the first webinar, and more results were added to the manuscript draft after the second webinar. SPC, single point of contact.

A process flow diagram depicted in Fig. 1 shall help to break down how we designed, planned, and executed our large-scale federated analytics study by depicting the flow of tasks in the five main domains (study conceptualization and data partner buy-in, protocol including the statistical analytical plan, ethical approval, analytical code, result interpretation, and result dissemination) across the study team. This process chart shall help the reader to navigate the complexities of a large-scale federated analytics study and to better benefit from the learnings and suggestions shared in the subsequent chapter.

## Study Execution: Key Learnings and Suggestions

In short, we considered (1) funding, (2) why it would be beneficial to use a large-scale multinational federated network analysis framework, we (3) defined the roles of the study team, (4) looked for participating data partners, (5) decided how to communicate, (6) decided on strategies to keep data partners engaged, (7) defined the timelines of the study, (8) created the analytical code to be executed by data partners, (9) decided on strategies how to interpret and (10) how to disseminate the results. We have detailed each of these steps, including our learnings and suggestions, in the following sections. To summarize these learnings, we established a summary checklist that lists key recommendations that shall guide future researchers in successfully navigating the complexities of large-scale federated network studies ( Table 2 ).

**Table 2:** Checklist for conducting successful large-scale multinational federated network studies

Topic	Check
1. Funding: We recommend joining an active and engaging community, where mutual collaboration drives the initiative, and data partners might engage for the greater good even when no funding is available. ➔ Look for funding opportunities to cover the data partner's expenses.	
2. Choose a relevant research question and approach: Large-scale federated analyses are a substantial commitment. ➔ Circulating and discussing the study idea widely with the community and interested parties will ensure that a relevant research question and approach are chosen.	
3. Attract buy-in: Larger networks yield more comprehensive, diverse, and generalizable evidence to inform clinical practice and policy decisions. ➔ Widely disseminate your study plans in community calls and conferences. ➔ Buy-in may be increased by branding your study.	
4. Establish clear communication channels: Creating a positive experience for the network through clear, empathic communication will align all participating data partners and encourage participation in future studies. ➔ Use a single point of contact to coordinate interactions with data partners, minimizing redundancy and confusion. ➔ Supplement emails with collaborative platforms (e.g., Microsoft Teams, GitHub) to foster engagement and address shared challenges efficiently. ➔ Give regular updates on where the study is and what is coming next to keep data partners engaged.	
5. Strategic division of responsibilities: People work best if there is a clear objective (and even more so if they are passionate about their tasks). ➔ Assign clear roles among the study team (lead/backup) and allocate the following responsibilities: • Project management • Communication • Protocol • Analytical code/data visualization tools • Data partner submissions • Webinar/Study-a-thon • Preprint/manuscript	
6. Set clear timelines: Time is crucial in any large-scale network study. Allow enough time but not too much. ➔ Define achievable deadlines for protocol feedback, code execution, result and ethical approval sharing, and sharing of administrative documentations, result discussions, and writing the study up into a preprint/manuscript. ➔ Update timelines if needed. ➔ Set a final deadline and stick to it.	
7. Phased rollout of analytical code: Avoid having to ask the participating data partner to re-run the code because it increases the workload and may reduce the positive experience. ➔ Dissect analytical code into logical chunks (feasibility, step 1, step 2, etc.) ➔ Use version control ➔ Pre-test study code with a subset of databases before full-scale deployment to identify and address potential issues early.	
8. Address data quality proactively: Better data leads to more true results. Make it a give-and-take experience for data partners by offering them insights into their data quality. ➔ Provide feedback on feasibility results for database mapping improvements. ➔ Offer tools like Shiny Apps for data visualization (supplemented by comprehensive documentation or a tutorial), which serve as local result validation and ultimately also for result dissemination (two in one)	
9. Community-based result interpretation and dissemination: Large-scale federated analyses leverage the community behind it and provide opportunities for shared learning, while leading to more meaningful evidence ➔ Engage data partners in interpreting results through virtual or face-to-face meetings. ➔ Invite data partners in the manuscript writing process to make the generated evidence richer	
10. Invite feedback for study quality improvement: Continuously improve your processes. ➔ Invite feedback from data partners at the end to improve your approach to large-scale federated analyses.	

### Funding

The EHDEN data partners agreed to participate in at least one network study as part of having the OMOP-CDM mapping of their data paid through the EHDEN subgrant agreement. Hence, there was no specific reimbursement for data partners to participate in our study.

The participation in most large-scale federated analytic studies within the OHDSI/EHDEN space had been voluntary, which highlights the importance of collaborative interaction within the community (please refer to the “Finding Data Partners and the Role of the Community in Federated Analyses” section). However, remuneration of the data partner's efforts may change on a study-to-study basis.

### Why Use Large-Scale Multinational Federated Network Analyses and the Observational Medical Outcomes Partnership Common Data Model?

To observe potential trends or differences in the use of medicines in relation to drug shortages across Europe in various health care settings does not have to be difficult when relying on established large-scale federated analytics networks. For example, drug formularies and clinical practice varies between countries, which is a challenge when assessing the multinational problem of drug shortages. However, the OMOP CDM serves as a “common language” shared by all data partners, enabling collaboration and interoperability across diverse databases and countries, and the use of standardized analytics generates robust and comparable results. Hence, we leveraged this framework for our goal to produce high-quality and meaningful evidence, and a large-scale multinational network analysis was the chosen approach to tackle the widespread and international problem of drug shortages in a standardized and timely manner.

### Study Team Structure

Large-scale multinational federated network studies do create more work than a smaller federated analysis (including at least two data partners). Thus, conducting one is a substantial commitment. Our study was co-led by M.P.M. and T.B. A co-lead administration was the ideal approach for our case, because it allowed us to split the work according to our strengths and preferences. One co-lead ensured the completion of the protocol (including a statistical analysis plan) and manuscript, evaluated the feasibility assessments, and followed up with data partners (M.P.M.). The other was on top of timelines, analytical R code, and visualizations (through Shiny Apps), resolving GitHub issues, and planning the webinars (T.B.). It is impossible to anticipate every little task right at the start of the project, but having a co-lead enabled us to distribute responsibilities, deliberate, and reach a consensus. This arrangement also ensured mutual support during holidays and periods of high demand caused by other concurrent projects. This strategy was complemented by a dedicated administrative staff member, who managed administrative tasks and served as the single point of contact for communications with data partners (M.C.); an additional administrative member that helped submitting the manuscript to the journal after the completion of the EHDEN funding; a senior analyst from our group that provided additional expertise for technical questions related to the analytical and visualization codes; a taskforce of engaged participants from EHDEN and EFPIA consortium members (European Federation of Pharmaceutical Industries and Associations) that provided input in monthly meetings and tested the code before its general release; the data partners themselves, including the manuscript team; webinar facilitators who helped the discussion into breakout groups; and an expert who handled external communications to update social media with our study progress. The individual tasks and processes, including all involved players, are depicted in the process flow diagram in Fig. 1 .

### Finding Data Partners and the Role of the Community in Federated Analyses

The community plays a vital role in federated analyses, which rely on the established network, mutual trust, and open communication channels. The relevance and applicability of the study question will influence the level of participation from data partners. The type (e.g., hospital, registry, or primary care data, etc.), completeness (e.g., availability of socioeconomic factors, mortality, specific treatments, etc.), and quality of data are key elements that affect the analysis and must be considered when selecting data partners. While all data partners need to pass OHDSI-inherent quality control checks before they are able to contribute to network studies, feasibility tests specific to the study aims are necessary to ensure the quality of the analysis.

Carrying out a study in a preexisting community or network increases the likelihood of receiving feedback and can help speed up the timelines for a study. EHDEN is part of OHDSI, and our study was executed solely within the EHDEN network of data partners and databases accessible to the EFPIA consortium. We invited data partners from the EHDEN network to participate when the first draft of the protocol was ready to share (month 0 in our timelines, Table 1 ). Intelligence from the data partners was necessary at this stage to make analyses as inclusive as possible by considering database or country-specific challenges. Out of the around 200 database custodians within the network, 92 expressed interest in joining, 67 handed in feasibility results, 63 passed the feasibility stage (i.e., to have at least one drug of interest recorded in more than 100 individuals within the study period), 54 handed in at least one set of aggregated results, but 2 did not share their ethical approval in time, and therefore, had to be excluded. Thus, we were able to process the results of 52 data partners.

The collaborative environment fosters a collaborative approach, where a researcher may lead one study and contribute as a data partner for another. The atmosphere in network studies is perceived as positive and welcoming, as participating data partners share the belief that results from federated analyses can make a significant impact on clinical practice. Being part of a large-scale multinational federated network study was described to amplify this sense of contribution due to its size and scope.

EHDEN and the broader global OHDSI community have been perceived as especially welcoming to new members, eager to share knowledge and provide training that enables them to lead their own network studies. In large-scale multinational federated network studies, many data partners may be joining for the first time. For the study leads, this means exposure to a great number of new databases and individuals, and the responsibility to maintain the good reputation of the community. For a new data partner, this offers an opportunity to connect and find support among fellow newcomers who may share similar experiences, and to learn from and with more experienced data partners.

### Communicating with Data Partners

We undertook a large communication effort to achieve a high level of participation from data partners. We campaigned our study through community calls and conferences, and a dedicated teleconference within the EHDEN network. Additionally, the academic lead and research coordinator of the EHDEN project championed this work. This generated strong interest and enthusiasm, making the study attractive to participants. At this time, the study was called “MegaStudy” for its ambitious size and scope. The name became a memorable brand that helped promote the study. Thus, it may be a worthwhile consideration for future project leaders to brand their study.

A crucial factor for successful communication for the execution of the study was having a single point of contact (M.C.) responsible for sending communications and receiving inquiries from data partners. Data partners valued the clear and empathic communication from the contact, especially regarding timelines, which kept everybody aligned. Furthermore, in addition to emails, a Microsoft Teams channel was used to amplify communications and promote a more engaging environment. Moreover, it helped minimize repetitive questions and strengthened the sense of community by enabling direct interactions among data partners. Microsoft Teams also served as a central repository for all documents, allowing version control and collaborative work on the same documents simultaneously.

### Keeping Data Partners Engaged

All data partners were encouraged to actively participate: At the protocol stage, through the Teams channel, discussing analytical code in the GitHub repository's issue tracker, discussing the results in the webinars, and by taking part in the manuscript writing or by commenting on the manuscript. Moreover, a small circle of EHDEN leads and EFPIA data partners were part of a task force with whom we had a monthly meeting (starting at around month −1, Table 1 ). This gave us the possibility to incorporate the participant's view into our strategy and planning. It not only made the study better and smoother, but it also gave us (self-set) monthly deadlines.

In every network study, the data partners will need to provide other information besides results. Some of which we captured in our tracker table ( Table 3 ): Data type, database management system, and database name used in the aggregated results. Others we requested were a short database description (including the approved database name for publication), ethical approval, author details, and a conflict of interest form for each individual. The most difficult part was—despite several reminders—to get the confirmation of ethical approvals from data partners, as well as a short database description, which we struggled with right until the upload of the first preprint. This part needed individual follow-up emails and was rather time-consuming. The fastest turnaround we had was for receiving author details and conflicts of interest information for the preprint. We advise collecting this information from data partners as early as possible to avoid delays in publication.

**Table 3:** Overview of our data partners tracking table that was located in the Teams channel and partially filled in by us (white background) and the data partner (gray background)

Number	Data partner	Country	Data type	Database name	DBMS	Feasibility	Analyses 1 Inc/Prev	Analyses 2 DUS	IRB/waiver
= Unique identifier (we just counted from 1 onwards)	= Name of the institution	= Name	Primary/Secondary/P + S	“As used in the analyses”	= Database management system	Awaiting results/Pending feedback/Pass/Not pass	Yes/Blank	Yes/Blank	Yes/Blank

Abbreviations: DBMS, database management system; DUS, drug utilization study; Inc/Prev, Incidence/Prevalence; IRB, Institutional review board (ethical approval), that is, ethical approval; P + S, primary and secondary care included in the data.

### Timelines

Timelines were continuously communicated and updated. To keep data partners informed, our communications included a flow chart showing the current study phase and the following steps, which was well-received. Table 1 shows the study timeline to serve as a reference for future projects. We found that aiming for a timeline of around 18 months from sending out the draft protocol (or 12 months from protocol registration) to the publication of results (at least as a preprint) proved efficient. The timeline depends strongly on the study's complexity, and ours was complex due to the number of assessed drugs and multiple objectives. To achieve this strict timeline, a structured project management approach was essential.

We structured our study in several phases equivalent to individual actionable tasks. We had to plan the order and the required time for each part, then we doubled our initial time estimations, and most importantly, we thought about risk mitigation strategies. The largest time period was allocated to run the analytical code (around 10 months for all data partners to hand in the results). The main mitigation strategy to ensure a large participation of databases was to keep enrolment open until month 14 (10 months after the protocol registration, and 4 months before study completion), as long as data partners uploaded their results and evidence of data sharing authorization (i.e., ethical approval) by month 15.

Many interested parties were new to OMOP and, at the beginning of the study, some were still finishing the data mapping and inherent quality control checks. Moreover, running the code could take from a few hours to several days, depending on the system executing the code, or weeks if they experienced issues. Thus, we had early deadlines for the early data partners and updated deadlines for those databases joining later in concordance with the protocol registration/webinars/preprint publications milestones ( Table 1 ). Accommodating a longer time period to hand in results was very much appreciated by data partners. We considered the inconvenience of incorporating the latest results in the preprint update and final manuscript to be worth the increased participation. Furthermore, having several small deadlines throughout the study helped to keep participants engaged. The completion of the study coincided with the ending of EHDEN's funding period, which created an additional sense of urgency that probably contributed to the rapid execution. In this context, data partners valued the flexibility of deadlines while understanding the necessity to eventually lock the data and complete the study.

Of note, we recommend being aware of region-specific special periods, such as summer or Christmas/New Year in Europe (but will differ in other continents), and to avoid deadlines within those periods.

### Analytical Code

The previous mapping of all data assets to the OMOP CDM was a critical step in enabling federated analyses. Additionally, the DARWIN EU analytics development team, as well as the HADES (Health Analytics Data-to-Evidence Suite) team from OHDSI, provided open-source, standardized analytical pipelines that are easy and ready to use for various analyses. 7 8 These pipelines consist of several R packages supporting every step, from concept selection and cohort creation to a wide range of analyses. The study code can be easily written by someone with basic knowledge of R. Moreover, the packages had been tested on the most common database management systems (MS SQL Server, PostgreSQL, Snowflake, Redshift, Databricks). However, we were unable to accommodate two data partners using Oracle and BigQuery.

At the protocol stage, we dedicated a good amount of time choosing a study design that could fit the variety of databases, and we asked data partners for feedback to confirm that planned analyses were theoretically applicable in their databases. This ensured that the transition from the protocol to results (via the analytical code) was smooth and time-efficient.

Additionally, there is the risk of losing data partners when code must be re-run. To ensure the successful execution of the code on the data partner's side, we applied the following mechanisms:

1. Use of a GitHub repository to ensure version control of the code: https://github.com/EHDEN/MegaStudy
2. Use of the renv R package to secure an identical R environment at the data partner's side to that in which the code was developed. R packages are constantly updated, and by the time data partners run code, the latest package version may not be compatible with other packages used in the code. Thus, securing the R environment is of utmost importance to secure a smooth rollout.
3. Having the code run successfully in-house by another person to verify that the code can be run by someone who did not write it, similar to what the data partners will do.
4. Having the code run successfully in a small circle of around five participating databases using the most frequent database management systems to capture problems that may arise when another system is used (and before dozens of data partners try it).
5. Asking those who experienced hiccups with the code to open GitHub issues to bundle similar problems, and give data partners the opportunity to help one another.
6. Providing a Shiny App to display results, enabling data partners to check their results before handing them in. Moreover, the Shiny App, including the results from all data partners, was later deployed as a standing web site, allowing everyone to explore the results as part of the result dissemination.
7. Providing individual feedback to each data partner based on the feasibility results to point out potential problems with the mapping of the drugs of interest and key related variables in the OMOP CDM (e.g., duration, quantity).
8. A progressive deployment of analytical code, which helped data partners to focus on just one code step at a time (feasibility: Using the DrugExposureDiagnostics package; analytical code 1: Using the Incidence/Prevalence package 13 ; and analytical code 2: Using the DrugUtilization package). We recommend basing the decision of splitting the analytical code into one or several steps, depending on the study question and the query size of the code. In our study, most data partners ran each code step within 1 to 2 months after its deployment. Some data partners joining later often ran all the code steps at once, by which time we had more experience with the code and could point data partners experiencing problems to prior GitHub issues that would resolve the matter.

Using the GitHub repository to ask questions and post issues was deemed very useful by data partners. They also appreciated that the analytical code was fully written and tested by the main leading team. However, some data partners faced challenges specific to local data environments, for not activating the renv R package (using older versions of R packages and/or due to dependencies of R packages), due to the large size of query, for using a different OMOP vocabulary version, and because the code needed to be re-run from the start if it failed. Thus, as a learning, we recommend saving the outputs throughout the analysis, rather than at the end of the script.

An important aspect for data partners participating in the study was receiving feedback on their own data. For example, the feasibility step feedback provided valuable insights into data quality and helped refine variables early in the study. Moreover, result visualizations through the provided Shiny Apps were used by data partners for local quality management, including discussing the results with local domain experts. While a more systematic and global quality assessment procedure was a wish for the future by some, it remains to be discussed whether this is the responsibility of the leading team. Other data partners took a more proactive approach and tackled the data quality head-on by investing a considerable time and resources to validate and assess the data themselves, which they considered essential for meeting the project's quality standards and also helped them to reduce efforts for the next study participation.

However, the Shiny app visualizations were not easily understood by all data partners, especially for those who were new to the tool. More detailed documentation would have helped them understand the results better before the first webinar, and potentially reduced their workload. Thus, offering a tutorial earlier than during the webinar would have been important to ensure participants could engage more effectively with the data and perform plausibility assessments in a timely manner.

### Results Interpretation

Every large-scale network study will inevitably accumulate a lot of results. In our study, results heterogeneity was expected across the various databases—not only between countries, but also across health care sectors—since medicine use and distribution vary. After having consulted with experts in the field, we chose a data-driven approach which made the number of results humanly manageable. Additionally, certain medications were more frequently used in primary care than in secondary care, and vice versa. Thus, we grouped the databases by data type when interpreting the results: Those containing primary care data, secondary care data, and others (including claims, mixed primary and secondary care, and registries).

All data partners were invited to be part of the results discussion, whether they had provided results or not, which were held in two virtual meetings (i.e., the webinars, one for each analytical code output). Data partners appreciated the webinars, which provided further explanations and facilitated discussion of the results with a focus on the outputs displayed in the Shiny App. In our opinion, webinars proved more efficient than an in-person study-a-thon because of the large number of participants..

It took around 3 months of planning for the first webinar, and 1 to 2 months of planning for the second webinar, as we could build on our experience. We suggest taking the webinars very seriously as there will be significant expectations from participants who have eagerly anticipated this opportunity to engage with one another. In Table 4 , we share the webinar agendas for inspiration. Gathering feedback through a quick online survey at the end of the first webinar helped us improve for the second webinar (in terms of timing: Longer breakout discussion; overall length: Having a shorter webinar; and making the Shiny app with all results available 1 week before the second webinar so that participants could familiarize themselves with the results). After each webinar, we made recordings available to those who wanted to catch up on the progress or review what had been discussed. Data partners gave the feedback that the meetings addressing specific topics enabled faster progress. In their opinion, this level of coordination helped maintain momentum, especially given the complexity and scale of the study. Furthermore, we share the opinion that the diverse backgrounds of participants were a further key to the webinar's success, as results were interpreted from various perspectives, allowing each participant to contribute their specific expertise. However, some data partners mentioned that they did not have a similar database to compare their results with, and that sharing of aggregated results required their organization to adapt to a new mindset. Yet, the timely and extensive feedback on study results helped them to build support by communicating with their internal and external stakeholders, and it also ensured their confidence in participating in future OMOP network studies.

**Table 4:** Time tables of our first and second webinars to discuss the results of our real-world evidence study containing 52 databases in 19 countries

First webinar	
9:30 hours	Opportunity for informal questions, talks, and getting to know one another with Marta and Theresa
10:00 hours	Welcome, introduction, and background (MegaStudy Team)
10:45 hours	Comfort break (5 minutes)
10:50 hours	Introduction to the Incidence/Prevalence Shiny App with the example of b/tsDMARDs and plenum discussion (co-lead, T.B.)
12:00 hours	Lunch break (1 hour)
13:00 hours	First breakout session in five groups (antibiotics) Group discussion (25 minutes) (Facilitators) Session Discussion (35 minutes) (Co-lead, T.B.)
14:00 hours	Comfort break (5 minutes)
14:05 hours	Second breakout session in five groups (other drug groups) Group discussion (25 minutes) (Facilitators) Session Discussion (35 minutes) (Co-lead, T.B.)
15:05 hours	Comfort break (5 minutes)
15:10 hours	From here, session—Discussion of next steps (Co-lead, M.P.M.)
16:10 hours	Closure (5 minutes)
16:15 hours	Informal get-together for questions, talks, anything (Co-leads)
Second webinar	
9:00 hours	Welcome, introduction, preprint, and Shiny App (Co-lead, T.B.)
9:45 hours	Comfort break (5 minutes)
9:50 hours	First discussion session of the four groups of drugs Group discussion (40 minutes) (Facilitators) Session Discussion (30 minutes) (Co-lead, T.B.)
11:00 hours	Comfort break (10 minutes)
11:10 hours	First discussion session of the three groups of drugs Group discussion (40 minutes) (Facilitators) Session discussion (30 minutes) (Co-lead, T.B.)
12:20 hours	Comfort break (5 minutes)
12:25 hours	From here, session—Discussion of next steps
13:00 hours	Closure

Abbreviation: b/tsDMARDs, biologic and targeted synthetic disease-modifying antirheumatic drugs.

### Results Dissemination

We used the momentum of the first webinar to create the manuscript team with the aim of helping us prepare the preprint. Thus, the work of manuscript writing and result visualizations was split between 20 participants who subsequently engaged in separate fortnightly meetings. The first discussion we had was about how many manuscripts to write and how to split the content because we had just so many results. Finally, we opted for one manuscript that would provide higher-level output by highlighting some examples. We split the team into several groups leading on a particular part of the manuscript (graphs, introduction, methods, results, discussion). A preprint was published on medRxiv in month 14, and the preprint was updated with more results (from the second webinar) in month 17. Having a preprint early helped because it gave a sense of achievement without the pressure that it had to be the best and final version, and with the early preprint, we were able to invite public review of our work. Results were also disseminated throughout the study at various conferences and at key EHDEN consortium meetings. Finally, all participants were invited to give feedback on the preprint, and the study team revised and formatted the preprint for submission to a scientific journal.

We limited the number of coauthors in the preprint and final manuscript to a maximum of three individuals per data partner (limited to active participation in the study, such as feedback, webinars, etc., and not to their availability to handle results). The order of the authors was determined as follows: The co-leads of the study, M.P.M. and T.B., were first and last authors, respectively. Coauthors that participated in the manuscript team were added in the middle alphabetically, except for D.P-A. and P.R., whose names were placed at the end, as senior leads of the EHDEN initiative. In the preprint, the remaining coauthors who participated as data partners were placed between the last manuscript team author and before DPA, also alphabetically ordered. Yet, some journals limit the number of authors, and therefore, “the remaining coauthors” were included as part of the “Drug Shortages EHDEN” group authorship.

## Conclusion

Our learnings and suggestions shall help other teams to perform successful large-scale multinational federated network studies within a timely manner. It is worthwhile undertaking large-scale multinational federated network studies because the additional evidence generation largely outweighs the additional amount of work compared with a smaller network study. The code is the same, as is the organization of files, and the timelines should be roughly the same. Very large-scale network analyses like this one just require additional effort of keeping up communications, best facilitated through a single point of contact, integration of the data partners' view through a task force, and smooth analytical code rollout through pretesting of study code by a subset of experienced data partners. Finally, the timely and successful conduct of large-scale federated analyses, as demonstrated here, resulted in positive experiences for data partners and their stakeholders and will hopefully allow and increase their participation in further network studies, which will ultimately feed sustainable large-scale evidence generation.

## ClinicalRelevance Statement

Federated analyses using the OMOP CDM, as demonstrated in our large-scale federated analyses network study, offer a powerful approach for generating real-world evidence across diverse health care systems. The scalability and reproducibility of such studies make them particularly relevant for addressing cross-border health care challenges with the potential to improve patient outcomes and clinical practice worldwide. However, as studies grow in size—such as ours, which included 52 databases—it becomes increasingly crucial to ensure high-quality standards. Adhering to best practices in planning, execution, and result interpretation is essential to uphold the integrity and reliability of findings, as the complexity and scale of these analyses demand meticulous coordination and robust methodologies. High-quality studies are foundational to maintaining confidence in the evidence generated and its application to inform clinical and policy decisions globally.

## Multiple-Choice Questions

1. What is one of the primary reasons federated network studies are advantageous for observational research?
  - They eliminate the need for analytical pipelines.
  - They allow data to remain within legal jurisdictions while enabling shared analytical insights.
  - They require fewer data partners to participate.
  - They bypass the need for data standardization.
  **Correct Answer** : The correct answer is option b. Federated network studies are advantageous because they maintain data within local legal jurisdictions while enabling shared analytical code and aggregated results across diverse health care settings. This ensures compliance with privacy laws and data governance standards while facilitating robust and reproducible research.
2. What was one key factor in ensuring the success of the large-scale federated analysis described in the study?
  - Avoiding the involvement of data partners with diverse expertise.
  - Using proprietary analytical pipelines for data analysis.
  - Establishing a single point of contact for communication with data partners.
  - Limiting engagement with data partners to reduce complexity.
  **Correct Answer** : The correct answer is option c. A single point of contact for communication was crucial for the success of the study. It streamlined interactions, provided clarity on timelines, and ensured alignment among all participants. This approach minimized confusion, reduced repetitive questions, and facilitated efficient problem-solving, contributing significantly to the project's timely execution and success.
